# International trainer perceptions of simulation-based learning: a qualitative study

**DOI:** 10.5116/ijme.61b3.214c

**Published:** 2021-12-27

**Authors:** Junichi Fukamizu, Daniëlle Verstegen, Sin Chi Ho

**Affiliations:** 1 Terumo Corporation, Tokyo, Japan; 2 School of Health Professions Education/Faculty of Health, Medicine and Life Sciences, Maastricht University, Maastricht Netherlands, the Netherland; 3 Terumo Asia Holdings, Singapore

**Keywords:** Simulation, facilitation, trust, feedback, continuing professional development

## Abstract

**Objectives:**

This study examined trainer perceptions of simulation-based learning for Continuing Professional Development in international settings.

**Methods:**

A qualitative research methodology was used to gain insight into trainer perceptions. Seventeen international physician trainers involved in simulation training in cardiovascular catheterization and intervention were interviewed. An inductive thematic analysis was performed following steps described by Braun and Clarke; researchers inductively approached, and then carefully dissected the transcripts into individual stories, grounded the problems, and explored themes.

**Results:**

Trainer perceptions are largely aligned with learning theories, even though they were not specifically educated in simulation-based learning and program design principles in advance. Trainers perceive their primary role as facilitators to be most important and consider structuring sessions, facilitating group learning, and stimulating reflection to be crucial themes in simulation-based learning. They believe that building trust is an underlying principle to function in their role and feel responsible for being prepared to improve trainee satisfaction as adult learners. Trainers believe that learning from making mistakes is an important mechanism in simulation-based learning, but they give less attention to giving feedback.

**Conclusions:**

Trainers with basic training in facilitation skills in a classroom may unconsciously follow teacher-student instructional models with which they are familiar. This study confirms that trainers in simulation-based learning need pedagogical and facilitating skills to guide trainees and facilitate group processes. Educational training for trainers should include building trust and giving feedback in a more explicit place. In future studies, a mixed-method methodology is suggested to evaluate multi-layered complexities of educational practices.

## Introduction

Simulation-based learning (SBL) has an important role in postgraduate education for healthcare professionals. However, the training is not always based on evidence-based learning theories and expertise. Trainers may not be well-prepared for their roles. There is little insight into how trainers perceive their role.

Demand for SBL in medicine has greatly increased due to concerns for patient safety as a consequence of the complexities of technological innovation in medicine.^1^ Recent innovation in minimally invasive therapies, such as cardiovascular catheterization and laparoscopic surgery, requires the acquisition of complex skills.^2^ For example, cardiovascular catheterization and intervention is a continuously and rapidly evolving specialty, and therefore, training must evolve to keep up with the changes. There are ethical concerns about honing the necessary skills while caring for patients.^3^ In the workplace, there is not enough opportunity to practice safely.  SBL provides a controlled environment where various scenarios can be recreated repeatedly without endangering patients.^4^ On top of social demands for patient safety, meeting the needs of individual learners is another reason to advocate SBL.^5^ SBL can be part of a more extensive curriculum^6^ in an educational institution or a specialty community, but this is not always the case.  Recent studies in Continuous Professional Development (CPD) clarify a lack of systematic curricula as an educational challenge^7^ and identify factors that make daily practice positive and negative.^8^ To build a CPD course, well-designed programs and skilled trainers are essential when conducting SBL.

Trainers can only fulfil their roles on the condition that there is a well-designed training program that is based on suitable learning theories.^9^^-^^13^ The primary role of trainers in SBL is that of facilitator. A facilitator is defined as a person “who facilitates learning, guides and stimulates reflection in the group of learners.”^14^ Simulation is a dynamic, ongoing, interactive process^15^ where learners project themselves in the minds and actions of others^16^ as if they are emotionally in the workplace and encounter a new situation.^17^ When a learner has difficulties understanding the situation or limitations of handling technical procedures, facilitators can help, discuss, stimulate, reflect, instruct, or rescue the learner to get back on the right track. According to Forrest et al., trainers in SBL programs need competencies such as (a) understanding specificities of simulators and being used to handle simulators, (b) prepare and provide a safe learning environment, (c) plan effective simulation learning instruction, (d) facilitation, (e) feedback and debriefing, (f) analytical debriefing by using recorded sessions.^18^  Burns believes in the importance of feedback during simulation-based learning and its situational adaptability to fit learners as an area of faculty development.^19^

Trainers are often subject-matter experts or industrial technical experts, but people in both of these groups often have limited training as facilitators. Some receive limited training in facilitation skills but not in underlying learning theories or design principles of SBL. Little is known about how they execute training and to what extent they understand and apply the learning theories that SBL is based. The purpose of this study is to gain insight into how trainers facilitate SBL, to understand their perceptions of their role as trainers in SBL and to determine to what extent these perceptions are in line with learning theories or design principles. The ultimate objective is to use these insights to better train trainers and improve SBL.

## Methods

### Study design

To get insight into trainer perceptions, we conducted individual interviews with subject-matter experts who were trainers in an SBL program. We used a purposeful sampling strategy to approach the trainers involved in SBL training addressing the management of complications during Percutaneous Coronary Intervention at an international conference.

International senior interventional cardiologists (ICs) were assigned to be trainers for the SBL session. The trainers managed a 105-minute session that consisted of three phases: (a) introduction: explaining the learning objectives to adjust trainee personal goals and case scenarios, (b) simulation: interactive, hands-on simulation including individual feedback and team debriefing at each station in a small group and (c) debriefing: group-debriefing and summarizing take-home messages in a large group. Junior ICs were the trainees. Four trainees formed a team and stayed at the same station to complete the hands-on simulation procedure, where each trainee had 15-20 minutes to be the first operator in pre-assigned scenarios. Sessions included 5 minutes of introduction,15 minutes of interactive case discussion, 70 minutes of hands-on simulation, and 15 minutes of debriefing.

### Participants

We approached trainers after the SBL sessions concluded at the Percutaneous Cardiovascular Revascularization (PCR) annual meeting on 21-23 May 2019. Seventeen trainers participated in the study (Table 1) with a median age of 50 years (range 35-60 years). The median experience was 24 years as a physician with 21 years of specialty practice in cardiology, and the median experience as an instructor in SBL was seven years (range 0-16 years). Sixty percent of the trainers stated they had no specific training in SBL theories or design principles. The remaining 40% of the trainers declared they only had the classroom training for facilitating learning. Study participants came from ten countries (Australia, England, France, Germany, Italy, Japan, Norway, Poland, Switzerland, and Thailand), and all were male. This study was approved by the Institutional Review Board of Terumo Corporation (Tokyo Japan) according to the guidelines for research in medicine involving the human, Japan Ministry of Education and the Japan Ministry of Health and Labour, ver. 2017 (HPE-2018-TIS01). All participants signed a written consent that explained the research project and its objectives, methods, procedure, and analysis. Data were anonymized.

### Data collection

A semi-structured interview guide was prepared. Interviews were conducted by the first author face-to-face (n=3) or using digital communication tools such as Skype, FaceTime (n=9) or telephone (n=5). Interviews for non-Japanese interviewees were conducted in English, and interviews with Japanese participants (n=4) were conducted in Japanese. Interviews were audio-recorded, and the median time of the interviews was 39 minutes (range 24-55 minutes). Conversations were transcribed literally, and the transcriptions were then verified by the interviewees. Interviews conducted in Japanese were transcribed in Japanese and translated to English after checking. The accuracy of findings was validated by (a) member checking and (b) triangulation, corroborating evidence from different individuals, types of data or methods of data collection.^20^

**Table 1:** Trainer characteristics (n=17)

Participant	Duration (Min.)	Experience (years)			Formal training	Gender	Age	Country
		Physician	Cardiology	Instructor				
No.01	36	20	15	3	N	M	46	Italy
No.02	30	15	13	4	N	M	41	Japan
No.03	40	25	21	15	N	M	51	Poland
No.04	39	36	31	10	Y	M	60	France
No.05	55	6	6	4	Y	M	37	Switzerland
No.06	40	26	26	6	N	M	51	Japan
No.07	54	21	10	7	N	M	44	UK
No.08	37	12	10	5	N	M	36	Austria
No.09	31	29	27	15	N	M	53	Japan
No.10	30	24	22	4	Y	M	50	Japan
No.11	24	32	28	10	N	M	56	UK
No.12	46	29	29	16	Y	M	56	Germany
No.13	45	21	17	0	N	M	44	Thailand
No.14	39	31	22	15	N	M	60	Norway
No.15	30	31	28	8	Y	M	57	France
No.16	39	6	6	5	Y	M	35	France
No.17	44	23	20	10	Y	M	49	Switzerland
Median	39	24	21	7			50	
Average	38.8	22.8	19.5	8.1			48.6	
					Yes-7	Male-17		
					No-10	Female-0		

### Data analysis

Researchers explore data to obtain a general sense of the data by reading the transcripts entirely several times, immersing themselves in the details, trying to get a sense of the interview as a whole before coding data or breaking it down into parts.^20^^,^^21^ An inductive thematic analysis was performed following steps described by Braun and Clarke: familiarizing yourself with your data, generating initial codes, searching for themes, reviewing themes, defining and naming themes, and producing the report.^22^ The analytic data group included three expert educators from Japan, Singapore, and the Netherlands.

## Results

Three themes emerged from the data: ‘Structuring the session’, ‘Facilitating learning as a group’ and ‘Stimulating reflection’. This section describes the trainers impressions concerning each theme.

### Structuring the session

Trainers feel strongly responsible for paying specific attention to the structure of the training session. They stated that an SBL setting differs from a classroom setting. They feel that repeated experience as a trainer helps them to improve their competency:

“Trainers need to be sharp, engage themselves in every manipulation, and structure the minds in the audience.” (No.17, Male, 49 years, Switzerland) 

Trainers feel responsible for optimizing resources in a limited timeframe and improving trainee satisfaction at the end of the session. They say that they can do this by setting clear goals for the session, forming small groups, and allocating clear roles for the trainees to achieve professional time management. They also emphasize the importance of explanations about the simulation model being used and its benefits and limitations. They describe how they use a whiteboard to list options, steps, and techniques, illustrate the complex schematic procedures in a simple manner, and report the group discussion.

“The whiteboard eventually becomes an elegant summary of the explanation of the logic behind the procedures. In case of time constraint due to trainee interest differs, time for self-practice is practically proposed.” (No.03, Male, 51 years, Poland)

### Learner characteristics

Trainers talk about adapting training to the characteristics and needs of trainees. This view is essential because there is a large variety of trainees who participate in this training. Trainers do not know what trainees exactly expect and need, and they only have one session with trainees so they need to grasp trainee characteristics in the first five minutes and tailor the session to the group, staying within the described goals of the training. To teach according to the characteristics of each trainee does not seem easy. When the difference in trainee experience hampers the process, the experienced trainees in a group could be used as educational resources. When a problematic trainee interrupts the session, trainers need to manage this and optimize learning for the whole group. One participant said:

“It is very difficult to manage different level of experience of learners. However, I still believe it is nice to have a different level of people. An experienced person can be utilized as a teaching resource.” (No.06, Male, 51 years, Japan)

Another participant stated:

“Grab the participant experience and personality such as pushy, a little demanding, or overconfident experienced person.” (No.11, Male, 56 years, UK)

#### Attitude to be prepared

Trainers express that both trainers and trainees need to prepare themselves before the training program. Trainers feel responsible for providing a valuable learning experience for trainees and being well prepared to have clear ideas about the goals they want to achieve and the methods they are going to use. Furthermore, they expressed that they need to be trained for this task:

“Try all the teaching points, options before the program. You can mistake, you can try what you want to try, but do not do it during the program.” (No.15, Male, 57 years, France)

In addition:

“The facilitator should be prepared. Otherwise not to be able to draw satisfaction easily because the level of the participants is high.” (No.10, Male, 50 years, Japan)

However, trainers stress that trainees need a certain level of preparedness, i.e., they need to have basic knowledge and skills, a positive attitude to teamwork and team learning. Trainers also expect that trainees could express constructive criticism during the group debriefing:

“Participants must be interactive with facilitators. It is mandatory.” (No.04, Male, 60 years, France)

Moreover,

“Participants need to master basic techniques in advance… participants need to have an interest.” (No.10, Male, 50 years, Japan)

In addition:

“Debriefing was a little superficial, only full of thank you.” (No.05, Male, 37 years, Switzerland)

#### Attitude to be open to new ideas

Trainers describe that to be a good trainer, they need knowledge about the procedures and how to deal with specific complications, but they also need to be aware of the fact that there is not always the best solution, and that they need to show trainees the need to be curious and explore different options. Curiosities trigger a research mind, where simulation can reproduce situations as a methodology of science.

“Taking on the number of places as a facilitator is a precious experience. Because a new theme comes out through the simulation. There is a desire to see more and to experiment more.” (No.09, Male, 53 years, Japan)

### Facilitating learning as a group

Trainers define their role as facilitators rather than instructors. They describe how they avoid giving direct instruction and instead stimulate thinking by asking questions.

#### Creating an open, festive atmosphere

Trainers try to create an open, festive atmosphere that leads to discussions and stimulates collaboration to learn from others in a group. Many different but similar ideas are reported: trainers talk about creating friendliness or keeping an open mind that could lead to good communication between trainees. Creating an open, festive atmosphere becomes key to be collaborative to learn in a group. Their experience is that, otherwise, it will be difficult because of many factors, including seniority and culture.

“Difficulty is participant ego, high-experienced person, stressful, embarrassing situation to demonstrate among many people and shy people.” (No.05, Male, 37 years, Switzerland)

#### Building trust

Trainers feel that it is part of their role as facilitator to develop a safe learning environment. Trainers stress that trust is a significant factor; it can accelerate or deaccelerate the process of learning. Their descriptions refer to trust at different levels: trainer-trainee, trainee-trainee, and trainer-trainer. One participant said:

“The facilitator also needs a sense of unity, trust and communication as a group.” (No.06, Male, 51 years, Japan)

Another participant said:

“If you ask questions to others, you can generate trust among the group.” (No.08, Male, 36 years, Austria)

Other participant expressed:

“Among the trainers (both facilitator and taskforce) need to have trust and experience to work with.” (No.13, Male, 44 years, Thailand)

One participant suggested that to promote building trust, a trainer book or coursebook could include the details about trainers, such as name, country, institute, portrait photo, speciality, scientific interests, or joys in life. This idea might save time if there is not enough time for icebreaking exercises during the training.

**Stimulating collaborative learning**

Trainers feel responsible for stimulating collaborative learning and expressing that sharing different ideas with other members is essential. They also say that learning to see what people do is also learning. In one training session, not all single trainees can complete the whole procedure; sometimes, another trainee takes over due to time constraints. Such situations also happen in the workplace when one physician does not finish the procedure. They stress that giving feedback aims not only at individuals but also to show that everyone is involved in each procedure and decision-making. The discussion eventually makes “thinking rise”. (No.02, Male, 41 years, Japan) One participant said:

“You may be able to learn more by asking questions to other learners that you could not notice alone.” (No.06, Male, 51 years, Japan)

Another participant said:

“One-to-one does not work very well. Learner stops thinking, looking for the right answer. Learner gets tense, the teacher gets directive and starts instructive.” (No.02, Male, 41 years, Japan)

#### Learning to work together

While developing individual response and team response is essential for patient safety in crisis management, trainers emphasize that building a team is critical, just like in clinical situations. They express: think as a team, call others, help, do not work alone; individual and team response to the crisis is critical. Increasing complexities of medical innovations and increasing patient safety demands, patient safety is the centre of medicine. One participant stated:

“Think as a team to seek what could be the best way to resolve the problem. Even less experienced people, nurses or technicians can also speak out the ideas how to deal with the problems.” (No.12, Male, 56 years, Germany)

Another participant said:

“To get out of the crisis, individual response and team response to the situation are both keys.” (No.07, Male, 44 years, UK)

### Stimulating reflection

Trainers feel responsible for guiding the trainees to reflect. They describe how they promote dialog by asking questions and advocate thinking aloud to stimulate prior knowledge and reflection. An interactive process of dialog or think-aloud stimulates not only the trainees but also trainers.

“My questions might stimulate you. your questions might stimulate me.” (No.08, Male, 36 years, Austria)

#### Learning from making mistakes

Trainers stress that trainees should be willing to explore and make mistakes. Discussing what went wrong can be an excellent way to stimulate reflection, but then nobody must judge things as right or wrong. They also keep in mind that it is about probability, not failure when trainees encounter difficulties. When one strategy does not go well, trainees can change to another strategy. There is no perfect single solution. However, most trainers also say that they often intervene when they go wrong and get them back on the right track. They are due to this mostly because of the limited timeframe.

“Let the participants try and mistake, explain why the thing goes wrong, why the thing goes well. Making mistakes is very educational.” (No.15, Male, 57 years, France)

#### Learning logic behind the procedures

Trainers describe that they should have robust knowledge about what they are going to teach. They refer to the procedures and algorithms of decision making in clinical situations, why and how it works, why and how it does not work. Trainers need this knowledge to anticipate different scenarios and stimulate discussion and reflection about different options, the balance between benefits and risks, on top of patient-specific considerations. Trainers should remember to discuss how to do things on technical aspects and listen to the ideas behind the plan and the actions.

“Participants want to know the how. The essential, however, is the why as the background of the how.” (No.14, Male, 60 years, Norway)

## Discussion

This study was undertaken to examine trainer perceptions of SBL. Although trainers were not specifically educated in simulation-based program design principles and educational theories in advance, their perceptions are largely aligned with design principles and learning theories. The results clearly show that trainers see themselves as facilitators rather than teachers, which is in line with the ideas behind SBL. Trainers perceive trainees as adult learners who are willing to be involved, learn through experiences, including making mistakes. Trainers carefully approach trainees as adult learners, opting to avoid direct instruction, if possible, and stimulating learning through experience. They take individual needs and interests into account when they structure the session and optimize trainee satisfaction at the end of the session. They become an instructor and intervene only when they feel that the intended learning goals will not be reached. Thus, trainers mainly take the role of facilitator and occasionally that of instructor.^14^ Trainers also feel responsible for stimulating collaborative learning and reflection because they think that those accelerate learning. They use different methods in line with experiential learning theory; for example, they ask trainees to think aloud,^23^ try and retry, and include learning from making mistakes.^24^ They put effort into stimulating group discussion at the end of the session, e.g., seeking logic behind procedures or discussing why it succeeded or not succeeded.

Trainers discussed relatively little about giving feedback to the trainees (other than stimulating reflection). It is, however, also an essential principle of SBL. Real-time verbal feedback helps perform complex tasks under high-cognitive load during SBL sessions and effectively improves the decision-making process.^25^^,^^26^ This might be because trainers perceive their role primarily as facilitators rather than as instructors. Also, they could be concerned that giving too much feedback during SBL scenarios would demotivate trainees (as suggested by Waitling and Ginsburg)^27^ and would not give the opportunity to make mistakes from which they could learn. Finally, since trainers are familiar with traditional instructional models that generate teacher-student interaction in classroom,^9^ they tend to follow that Frenk and colleagues categorize this mindset as former generations of educational reforms.^28^

A new generation of education reform may be required that adopts core professional competencies in the workplace that has complex interactions by emphasizing the importance of feedback, such as physical materials including drugs, devices or organs, tissues, responding to described rules, regulations, guidelines, or non-verbally described culture, symbols, in addition to social, emotional relations with others. Giving feedback could then create meaningful and interactive SBL.

Building trust was an important topic during the interviews, but this is not explicitly mentioned in design principles established for SBL. The trainers see that trust is an underlying concept for all other design principles to function. Without trust, trainees may fear failing; they may not listen to feedback and reject learning as a team. Trainers extend the idea of building trust among trainers-trainees to among trainees-trainees and trainers-trainers. As trainers advocate that building trust is the central theme when executing SBL in an international setting, it suggests that trust may be significantly vital in SBL sessions where trainers do not know one another and need to work with foreign technical experts. This study has helped to gain insight into how this collaboration as a pioneer attempt of academia-industry partnering in CPD works when all the stakeholders and new frontiers including government, industry, media, advocacy, public or malpractice, certification, accreditation are involved in playing roles for patient care those should be integrated into a healthcare system.^29^ Hence, it is observed that when the learning environment becomes more cross-national, cross-disciplinary, cross-cultural settings, building trust will be more highlighted as the central concern.

Another new principle that trainers talk about is teaching trainees how they need to collaborate as a team in daily practice and encouraging trainees to work as a team in crisis even for individual, technical, complex skill acquisition as primary objectives. Learning collaborative work relates to patient safety, as Bleakley criticized that paramount education theories lack paying attention to the team power.^30^ Hodges recently stressed that clinical practice becomes more inter-professional and team-based.^31^ Although team skills are usually only stressed for situations where the scenarios focus on team functioning or non-technical skills, and the study implicates that team skills should be added to the design principles even in a technical driven SBL.

Although we used accepted qualitative research methods, methodological limitations may affect these findings. The first limitation of this study was that the participants were limited to a purposeful sampling of trainers. A cross-disciplinary approach is preferred for future research, including trainees and organizers, to get insight into their perspectives.^20^ The sampling was also restricted to a potential pool of 20 trainers at the congress. However, since 17 out of 20 (85%) of the trainers participated, and they represent different organizations and countries, the validity of the data could still be strong. The second limitation was that the study setting was limited to CPD training organized by a specific academia-industry collaboration for cardiovascular training. This specific academia-industry collaboration still has advantages because the planning group consisted of representatives of both academia and industry, who could routinely discuss or share missions and values, align goals and methods, adjust the process to goals, and leverage the strength of one another. The third limitation of this study is that data is limited to the perceptions of trainers and do not consider other indicators to measure. However, as the participants expressed, the research interview allowed them to stop and reflect, which generated their ownership of the project. Lastly, the data analysis may have been hampered by the difference in languages and cultural backgrounds in a cross-national research environment. In the analysis process, we realized the ways of thinking were influenced by traditions of values or sociocultural contexts in each community they belonged.^32^ We listened to the interview recordings repeatedly to be familiar with what they spoke about, inductively approached, and then carefully dissected the transcripts into individual stories, grounded the problems, and explored themes.

In future studies, a mixed-method methodology may enable evaluation of multi-layered complexities of educational practices^33^ in order to examine how SBL  can change daily practices in the workplace and analyze why the socioeconomic impact or patient-related outcomes remain uncertain.^34^

## Conclusions

The results of this study demonstrate that the perceptions of international simulation trainers are largely aligned with learning theories, even though they were not specifically educated in simulation-based learning and program design principles in advance. Trainers perceive their primary role as facilitators to be most important and consider structuring sessions, facilitating group learning, and stimulating reflection to be central themes in SBL. Trainers believe that building trust is an underlying concept to function in their role and feel responsible for being prepared to improve trainee satisfaction as adult learners who like to be involved in learning situations.

There are several implications for using these insights to better train trainers and improve SBL in the future. First, these results show that SBL programs must pay attention to team skill development and crisis management, stimulating trainees to learn from making mistakes. Building trust needs a more explicit place in the design of SBL programs. Second, these results suggest that trainer development in SBL needs to highlight the importance of feedback. Trainers, with basic training in facilitation skills in a classroom may unconsciously follow static teacher-student instructional models with which they are familiar. Trainers in simulation-based learning need pedagogical and facilitating skills to guide trainees and facilitate group processes. Educational training for trainers should include building trust and giving feedback in a more explicit place.

### Acknowledgement

Olivier Muller and international simulation trainers, for their devotion to the joint educational project and participation in the study; Lucas Lauder, for program evaluation and analysis of euroPCR2018; William Wijns, for insight, inspiration on simulation-based learning and Magali Breheret, for consistently monitoring the process to the planned goal of the joint educational project.

### Conflict of Interest

The authors declare that they have no conflict of interest.
